# OBJECTIVE SLEEP AND CIRCADIAN REST–ACTIVITY RHYTHMS AND MULTIPLE DOMAINS OF COGNITIVE FUNCTION IN OLDER ADULTS

**DOI:** 10.1093/geroni/igad104.1287

**Published:** 2023-12-21

**Authors:** Katie Stone, Haley Barnes, Terri Blackwell, Nancy Glynn, Karyn Esser, Anne Newman, Steven Cummings, Peggy Cawthon

**Affiliations:** California Pacific Medical Center, San Francisco, California, United States; California Pacific Medical Center, San Francisco, California, United States; California Pacific Medical Center, San Francisco, California, United States; University of Pittsburgh School of Public Health, Pittsburgh, Pennsylvania, United States; University of Florida, Gainesville, Florida, United States; University, Pittburgh, Pennsylvania, United States; California Pacific Medical Center Research Institute, San Francisco, California, United States; California Pacific Medical Center, Research Institute, San Francisco, California, United States

## Abstract

Evidence suggests that sleep and circadian rest-activity rhythms (RAR) play a key role in dementia and Alzheimer Disease (AD) pathology. Studies with objective measures of sleep and RAR and multiple domains of cognition remain limited. We studied 820 older adults enrolled in the Study of Muscle, Mobility and Aging (SOMMA). Actigraphs were worn for one week, and processed for sleep-wake measures including total sleep time (TST) and sleep efficiency (SE); and parametric (acrophase, pseudo-F) and non-parametric (interdaily stability [IS], intradaily variability [IV], and relative amplitude [RA]) 24-hour RAR variables. Cognitive assessments included global cognition (the Montreal Cognitive Assessment), executive function (Trails B test), memory (California Verbal Learning Test-short form; and psychomotor performance (Digit-Symbol Coding Test). Adjusting for age, sex, clinic site and education, we examined adjusted mean cognitive test scores by categories of sleep and RAR exposures. Mean age was 76 years, 58% were female, and 15% were non-white. Higher RA, reflecting a more robust 24-hour RAR, was associated with better cognition on all tests (p for trend <0.05). Reduced SE was related to lower cognitive function (psychomotor performance, global cognition, memory; p<.05). There were no associations with TST and any cognitive measures examined. SE and RAR were variably associated with both global and specific domains of cognition in older men and women. The RA in particular was strongly related to multiple domains of cognition related to AD, and may be useful in identifying individuals at risk. Additional analyses will explore sex differences and longitudinal associations.

